# Intensive Care in Sub-Saharan Africa: A National Review of the Service Status in Ethiopia

**DOI:** 10.1213/ANE.0000000000005799

**Published:** 2021-11-07

**Authors:** Fitsum Kifle, Yared Boru, Hailu Dhufera Tamiru, Menbeu Sultan, Yenegeta Walelign, Azeb Demelash, Abigail Beane, Rashan Haniffa, Alegnta Gebreyesus, Jolene Moore

**Affiliations:** From the 1 College of Medicine, Department of Anesthesia, Debre Birhan University, Debre Birhan, Amhara, Ethiopia; 2 Network for Perioperative and Critical Care (N4PCc), Ethiopia; 3 Department of Emergency Medicine and Critical Care, ALERT Hospital, Addis Ababa, Ethiopia; 4 Medical Service Directorate General, Ministry of Health, Addis Ababa, Ethiopia; 5 Harvard T.H. Chan School of Public Health, Boston, Massachusetts; 6 Department of Emergency Medicine and Critical Care, St. Paul’s Hospital Millennium Medical College, Addis Ababa, Ethiopia; 7 Emergency and Critical Care Directorate, Ministry of Health, Addis Ababa, Ethiopia; 8 Mahidol Oxford Tropical Medicine Research Unit, Bangkok, Thailand; 9 Institute for Education in Medical and Dental Sciences, School of Medicine, Medical Sciences and Nutrition, University of Aberdeen, Aberdeen, United Kingdom.

## Abstract

The burden of critical illness in low-income countries is high and expected to rise. This has implications for wider public health measures including maternal mortality, deaths from communicable diseases, and the global burden of disease related to injury. There is a paucity of data pertaining to the provision of critical care in low-income countries. This study provides a review of critical care services in Ethiopia.

**METHODS:**

Multicenter structured onsite surveys incorporating face-to-face interviews, narrative discussions, and on-site assessment were conducted at intensive care units (ICUs) in September 2020 to ascertain structure, organization, workforce, resources, and service capacity. The 12 recommended variables and classification criteria of the World Federation of Societies of Intensive and Critical Care Medicine (WFSICCM) taskforce criteria were utilized to provide an overview of service and service classification.

**RESULTS:**

A total of 51 of 53 (96%) ICUs were included, representing 324 beds, for a population of 114 million; this corresponds to approximately 0.3 public ICU beds per 100,000 population. Services were concentrated in the capital Addis Ababa with 25% of bed capacity and 51% of critical care physicians. No ICU had piped oxygen. Only 33% (106) beds had all of the 3 basic recommended noninvasive monitoring devices (sphygmomanometer, pulse oximetry, and electrocardiography). There was limited capacity for ventilation (n = 189; 58%), invasive monitoring (n = 9; 3%), and renal dialysis (n = 4; 8%). Infection prevention and control strategies were lacking.

**CONCLUSIONS:**

This study highlights major deficiencies in quantity, distribution, organization, and provision of intensive care in Ethiopia. Improvement efforts led by the Ministry of Health with input from the acute care workforce are an urgent priority.

**KEY POINTS**

- **Question:** What is the status of intensive care provision structure, organization, workforce, resources, and service capacity in Ethiopia?
- **Finding:** There is limited intensive care bed availability and distribution, and basic (oxygen, monitoring) and advanced (organ support, invasive monitoring) resource shortages are widespread.
- **Meaning:** Intensive care in Ethiopia is inadequate, and there is a need to improve the number, capacity, and services to enhance critical care provision.

**See Article, p 926**

Despite remaining one of the lowest-income countries in the world, Ethiopia has continued to improve its health status over the last 2 decades. The life expectancy of its citizens has improved from 45 years in 1990 to 64 years in 2014; maternal mortality ratio has declined from 1400 deaths per 100,000 live births to 351 in 2016 and under-5 mortality rate has declined by 67%, to 68 deaths per 1000 births. These results were achieved as a result of strong commitment from the Ethiopian government as well as technical and other resource support from development partners.^1,2^ However, until recently, little investment has been made into secondary and tertiary care services.

Although childbirth and infectious diseases remain the primary causes of mortality, emergency care and surgical burden, notably related to road traffic collisions are rising.^3,4^ To address the significant surgical burden, improvements in surgical access have been addressed through national programs, with a 10-fold increase in the number of surgeries per 100,000 people from 2012 to 2019.^5^ An accompanying expansion of critical care services has not been demonstrated and there remains a paucity of data pertaining to the provision of critical care in Ethiopia, as in many low- and middle-income countries (LMICs).

The concept of an intensive care unit (ICU) in Ethiopia began in 1956 at the Leelit Tsehay Hospital, yet it was another 37 years before a second ICU specifically for treating septic abortion and malaria was established. Currently, 53 public hospitals in Ethiopia provide intensive care services. To date, there has been no coordinated effort to evaluate critical care capacity in the country. Knowledge and understanding of the current status of services are the first step in planning, developing, and expanding the services.

This survey of ICUs across Ethiopia provides an overview of the baseline status of the country’s critical care service, including structure, staffing, equipment, and academic activities, to identify needs and priority focus for scale-up. This is the first comprehensive review of publicly funded ICU services in Ethiopia.

## METHODS

A multicenter hospital-based assessment was conducted using a structured survey over 2 weeks from September 1 to September 14, 2020. The study was approved by the Institutional Review Board (IRB) of Debre Birhan University (protocol number 04/2020-P004). Permission for data collection at each facility was requested and obtained by the Ministry of Health (MOH), who were key collaborators on this project. Verbal consent was taken from any staff providing information at a site level. The requirement for written informed consent was waived by the IRB. This article adheres to the applicable Strengthening the Reporting of Observational Studies in Epidemiology (STROBE) guidelines.^6^

The survey tool (available on request) was developed using published surveys in LMICs^7–9^ and included questions covering the 12 variables proposed by the World Federation of Societies of Intensive and Critical Care Medicine (WFSICCM) taskforce.^10^

1. Availability of skilled medical personnel
2. Availability of skilled nursing personnel (we utilized nurse to patient ratio)
3. Availability of other specialists: respiratory therapists, physiotherapists, nutritionists, etc
4. Capacity to monitor acutely ill patients
5. Availability of resources for the support of failing organ function
6. Design and structure of the physical space
7. Integration with ICU outreach services—in the emergency department and hospital ward, as well as services for follow-up of discharged patients
8. Presence of formal educational and professional development services for staff
9. Presence of dedicated house staff and role as a center for training expert personnel
10. Capacity for research and quality improvement activities
11. Role in acting as a referral service for the hospital, the community, and the country
12. Ability to scale-up services in response to a natural or human-made disaster or pandemic outbreak.

Twenty-two data collectors, who were nursing and medical staff with more than 2 years clinical experience in critical and emergency care, were recruited through the Ethiopian MOH. All data collectors received a 1-day orientation on the survey tool and were subsequently deployed to MOH-registered public facilities for data collection. The survey tool included face-to-face interview questions of ICU representatives supplemented with narrative discussions with hospital administrative staff as well as onsite assessments of individual ICUs. This 2-step process was conducted for all items within the survey tool. Where any discrepancies were identified, further validation of data was conducted by telephone call or site visit (where proximity made this feasible) by a study coordinator to ensure accuracy of data. Any data items which could not be verified were recorded as “missing.” Data collectors did not collect data from their usual workplace and study coordinators traveled to selected sites to supervise the data collection process. No individual identifiers were collected.

According to WFSICCM, level 1 ICUs are capable of providing oxygen, noninvasive monitors, and clinical nursing care that is more comprehensive than a ward, while level 2 ICUs can provide invasive monitoring and basic life support. A level 3 ICU provides a full spectrum of monitoring and life support technologies, serves as a regional resource for the care of critically ill patients and may play an active role in developing the specialty of intensive care through research and education. Each of the 12 variables was classified level 1, 2, or 3 as proposed by WFSICCM.^10^

### Setting

Ethiopia consists of 10 regions and 2 administrative cities. The regions are Oromia, Amhara, Tigray, Southern Nations Nationalities and Peoples Region (SNNPR), Sidama, Somali, Afar, Benishangul-Gumuz, Gambella, and Harari regions. The 2 administrative cities are Addis Ababa and Dire Dawa. Ethiopia has a population of 114 million people. The capital Addis Ababa, located in the center of the country, has around 4 million people (4.5%). Ethiopia is the second-largest country in Africa by population, with much of the population living in rural areas, with agriculture being the most promising resource for the region, comprising over 40% of gross domestic product (GDP), 60% of exports, and more than 80% of total employment. Ethiopia’s economic growth has averaged 9.9% year-on-year for 10 years since 2008. In recent years, this steady growth has attracted foreign investment, a feat very few African countries have achieved.

According to the MOH’s Emergency and Critical Care Directorate, 53 public hospitals with ICUs were providing ICU services during the study period. As a result of the pandemic, 2 of these units, based in Addis Ababa, provided only coronavirus disease 2019 (COVID-19) patient care and were closed for other services; therefore, these units were not included in the study.

This was a pragmatic study of all public hospital ICUs nationally registered by the MOH, with the exception of the 2 units treating COVID-19 exclusively. Data were analyzed using Stata 15.0 and are presented descriptively.

## RESULTS

There were 51 ICUs (Figure) and 324 ICU beds for a population of 114 million people, giving Ethiopia a national ICU bed to population ratio of 0.3 to 100,000. The distribution of ICUs within the country varied by region (range 1 to 11 ICUs per region; Table 1). The capital city, Addis Ababa, had 81 ICU beds, 1 quarter (25%) of the country’s ICU bed capacity. All hospitals in Addis Ababa had ICU bed capacity, ranging from 1% to 6% of Addis Ababa’s total hospital bed capacity. Outside of Addis Ababa, regional ICU capacity is <1% of the region’s total hospital bed capacity.

**Table 1.** Regional Distribution of ICUs and Beds

Region	Hospitals with ICU	Total beds (range/ICU)
Addis Ababa	10	81 (3–14)
Oromia	11	56 (3–10)
Amhara	9	63 (4–14)
SNNPR	8	38 (3–8)
Tigray	3	34 (4–24)
Sidama	3	17 (4–8)
Benishangul-Gumuz	2	8 (4)
Afar	1	6 (6)
Dire Dawa	1	5 (5)
Gambella	1	2 (2)
Harari	1	10 (10)
Somali	1	4 (4)
Total	51	324 (3–24)

Abbreviations: ICU, intensive care unit; SNNPR, Southern Nations Nationalities and Peoples Region.

**Figure.**
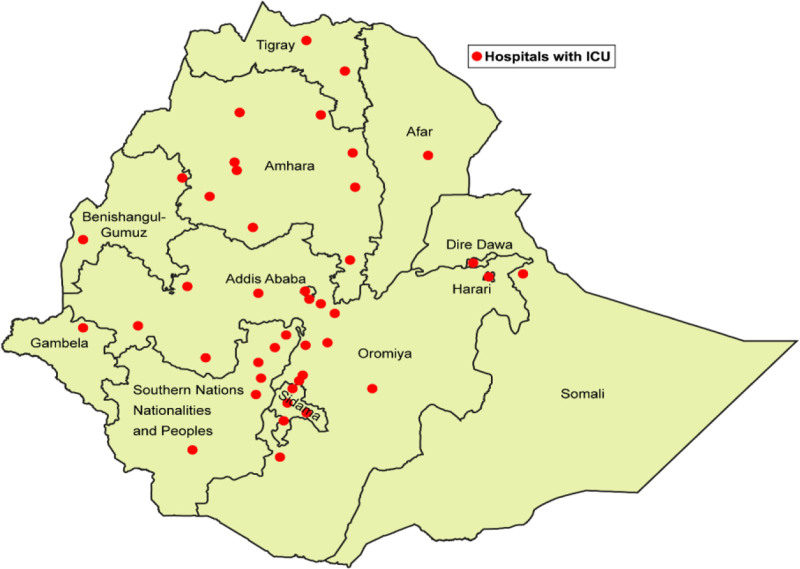
Location of Ethiopian ICUs. ICUs indicates intensive care units.

No ICUs had piped oxygen. In total, 276 (85% [95% confidence interval (CI), 81–89]) ICU beds had an oxygen cylinder, 205 (63% [58–69]) had a suction machine, and 106 (33% [28–38]) had all of the 3 basic recommended noninvasive monitoring devices (sphygmomanometer, pulse oximetry, and electrocardiography).

A total of 203 (63% [95% CI, 57–68]) ICU beds had functional ventilators. Only 9 (3% [1–5]) were equipped with invasive monitors. A medication infusion pump was available for 285 (88% [84–91]) of the ICU beds. Defibrillators were available in 37 of 51 (73% [58–84]) of ICUs, only half (n = 26; 51% [37–65]) of ICUs had a resuscitation trolley and 9 (18% [8–31]) had access to a portable x-ray machine. Only 5 (10% [3–21]) had access to renal dialysis at the facility and no ICU had capacity for continuous hemofiltration. A summary of resources and equipment in facilities is shown in Table 2.

**Table 2.** Provision of Equipment in Ethiopian ICUs

Equipment	Availability in the hospital (n = 51), n (%) [95% CI]	Availability of functional equipment per ICU bed (n = 324), n (%) [95% CI]
Electrocardiogram	49 (96) [87–100]	310 (96) [93–98]
Sphygmomanometer	49 (96) [86–100]	315 (97) [95–99]
Pulse oximeter	51 (100) [93–100]	321 (99) [97–100]
Thermometer	40 (78) [65–89]	250 (77) [72–82]
End-tidal monitoring device	21 (41) [28–56]	142 (44) [28–50]
Invasive monitoring devices	10 (20) [10–33]	9 (3) [1–5]
Oxygen cylinder	51 (100) [93–100]	276 (85) [81–89]
Oxygen pipeline	0 (0)	0 (0)
Mechanical ventilator	43 (84) [71–93]	203 (63) [57–68]
Infusion pumps	42 (82) [72–93]	285 (88) [84–91]
Syringe pumps	41 (80) [67–90]	293 (90) [87–93]
Suction machine	51 (100) [93–100]	191 (59) [53–64]

Abbreviations: CI, confidence interval; ICU, intensive care unit.

The design and structure of ICUs in Ethiopia vary; less than half (n = 23; 45% [95% CI, 31–60]) of units were located in proximity to operating, emergency, and imaging rooms. Only 3 (6% [1–16]) of the facilities with ICUs had a dedicated budget for critical care consumables, staffing, maintenance, and education. Around 3 quarters (n = 37; 73% [58–84]) of facilities had admission and discharge policies.

Staffing and specialty workforce was similarly higher in the capital city, with 37 of 72 (51% [95% CI, 39–63]) critical care physicians and 260 of 711 (37% [33–40]) critical care nurses located in Addis Ababa. In other regions, not all ICUs had critical care physicians. Medical specialist staffing included 27 anesthesiologists (38% [26– 50]), 13 (18% [10–29]) emergency and critical care physicians, 17 (24% [14–35]) neurologists, 6 (8% [3–17]) pulmonologists, 5 (7% [2–16]) intensivists, and 4 (6% [2–14]) cardiologists. Senior medical personnel were available 24 hours per day and could be present within 30 minutes. Treatment plans for new admission were discussed with, and ward rounds led by these senior personnel in 45 (88% [79–96]) facilities, although they were not always available for twice daily ward rounds (n = 35; 69%; [54–81]). All units had access to other specialties for consultation (medicine, surgery, and anesthetics), 38 (75% [60–86]) had an ICU director and 41 (80% [67–90]) a nurse director. Nurse to patient ratio was not <1:2 in most units (n = 46; 90% [79–97]). Pharmacy services were available in 28 (55% [40–69]) units. An established ICU committee was present in only 8 (16% [7–29]). None of the ICUs had an outreach service. Seven ICUs (14% [6–26]) had regular academic activities for their staff, and most (n = 38; 75% [60–86]) had no research or quality improvement activity.

Not all facilities were able to provide information on infection prevention and control (IPC). Only 22 of 47 (47% [95% CI, 32–62]) of units had access to a focal person for IPC. Seven (14% [6–26]) had a designated room for isolation. Less than a quarter (n = 10; 20% [10–34]) of units had policies for patient isolation, visitation, and control of traffic, that is, had designated times for visiting and restrictions on the number of visitors. Only 19 of 45 (42% [28–58]) provided training on IPC for the unit staff and only 9 of 45 (20% [10–35]) monitored infection rates. Handwashing facilities were available in 30 of 43 (70% [54–83]), clean and dirty utility rooms in 22 of 47 (47% [33–62]), a separate room for patient food preparation in 10 of 41 (24% [12–40]), and separate facilities for family members in 6 of 46 (13% [5–26]). The health care providers had access to personal protective equipment (PPE) in 32 of 45 (71% [56–84]) of the facilities which included facemask, face shield, gloves, and gowns. Nineteen units (of 47; 40% [26–56]) reused endotracheal tube suction catheters. Antibiotic stewardship strategies and strategies to reduce the use of antibiotics associated with clostridium difficile infection were in place in only 7 of 45 (16% [7–30]).

Utilizing the recommendations for classification of each of the 12 variables as proposed by the WFSICCM taskforce,^10^ each variable was categorized for each ICU in our study (Tables 3 and 4). For 3 domains—capacity to monitor ill patients, resources for support of failing organ systems, and ICU outreach—no ICU met level 3 criteria. In 7 of the 12 variables, more than half of all facilities met only level 1 classification.

## DISCUSSION

This study provides a review of critical care services in Ethiopia. Inadequate critical care has implications for wider public health measures including maternal mortality, deaths from communicable diseases, and the global burden of disease related to injuries. The burden of critical illness in LICs is high,^11^ with evidence that patients admitted to ICU’s are younger. Common reversible reasons for admission include sepsis, trauma-related head injuries, obstetric complications, surgical emergencies, and communicable diseases.^8,12–14^

**Table 3.** Categorization of Facilities for Each of the WFSICCM Variables 1–6

Variable, level, and criteria		No. facilities n (%)
Availability of skilled medical personnel		
1	Physicians with some critical care experience at least during the day. Variable access to other specialists	7 (14)
2	Physicians with ICU training/experience availab le during day and night. Ready access to other specialists	29 (57)
3	Physicians with formal ICU training 24/7. Rapid access to full complement of specialists	15 (29)
Nurse to patient ratio		
1	Higher than ward nurse to patient ratio	20 (39)
2	Not <1:3	8 (16)
3	Not <1:2	23 (45)
Availability of other specialists—respiratory therapists, physiotherapists, nutritionists, etc		
1	Other personnel available	22 (43)
2	Variable inclusion of allied health personnel	21(41)
3	Allied health personnel as regular team members	8 (16)
Capacity to monitor acutely ill patients		
1	Noninvasive or minimally invasive monitoring	37 (73)
2	Invasive (blood pressure, central venous pressure), blood gas analysis	14 (28)
3	Advanced hemodynamic monitoring (ultrasonography, cerebral, etc)	0 (0)
Availability of resources for the support of failing organ function		
1	Capacity for oxygen therapy and noninvasive organ support	46 (90)
2	Basic mechanical ventilatory and pharmacological cardiovascular support, intermittent RRT, nutrition	5 (10)
3	Advanced ventilatory and hemodynamic support, continuous RRT, tracheostomy	0 (0)
Design and structure of the physical space		
1	Dedicated geographical area	40 (78)
2	Dedicated area with central monitoring station	10 (20)
3	Dedicated area, individual patient areas, and central monitoring station	1 (2)

Variables and corresponding criteria adapted from the WFSICCM proposed classification of ICUs.^10^

Abbreviations: ICU, intensive care unit; RRT, renal replacement therapy; WFSICCM, World Federation of Societies of Intensive and Critical Care Medicine.

**Table 4.** Categorization of Facilities for Each of the WFSICCM Variables 7–12

Variable, level, and criteria		No. facilities n (%)
Integration with ICU outreach services		
1	Defined geographical area only	0 (0)
2	Ad hoc interactions with other care areas	51 (100)
3	Outreach team, step-down, close collaboration with other care areas	0 (0)
Presence of formal educational and professional development services for staff		
1	Variable engagement in continuing education	31 (60)
2	Engagement in continuing education	13 (26)
3	Regular engagement in continuing education	7 (14)
Presence of dedicated house staff and role as a center for training expert personnel		
1	Experienced nursing care 24/7. Ad hoc educational activity	43 (84)
2	Nurses with extra training in critical care provide 24/7 care	7 (14)
	Organized educational activities for staff	
3	Nursing staff with specialist ICU training provide 24/7 care	1 (2)
	Formal educational program for staff	
Capacity for research and quality improvement activities		
1	Basic quality improvement program	39 (77)
2	Formal quality improvement program. Ad hoc research	8 (16)
3	Formal education and quality improvement program. Active research	4 (8)
Role in acting as a referral service for the hospital, the community, and the country		
1	Ad hoc. Policy for transfer to higher ICU	0 (0)
2	Resource for critical ill patients within hospital	20 (39)
3	Referral resource for other hospitals	31 (61)
Ability to scale-up services in response to disaster or pandemic outbreak		
1	Responsive in disaster	32 (63)
2	Resource for critical ill patients within hospital	16 (31)
3	Disaster preparedness plan and capacity	3 (6)

Abbreviations: ICU, intensive care unit; WFSICCM, World Federation of Societies of Intensive and Critical Care Medicine.

Critical care capacity in LMICs remains largely unreported, particularly in sub-Saharan Africa.^15^ A 2020 review of critical care in the African continent reported an average of 0.3 ICU beds per 100,000 people. The number of ICU beds and ventilated beds in Ethiopia was reported as 570 and 557, respectively, but appears to have been estimated based on media reports.^16^ The current, on-the-ground study reports a lower figure with 0.3 public ICU beds per 100,000 population. Although higher than the rate of 0.1 reported for Uganda,^17^ this is far lower than the 8.9 previously reported in South Africa^12^ and inadequate to meet the needs of Ethiopia. A recent study revealed most sub-Saharan African countries (excluding Island nations) have <2.0 critical care beds per 100,000, with the exception of Eswatini, Gabon, Namibia, and South Africa.^18^ We report a bed capacity in Ethiopia consistent with this study, higher than Uganda (0.1), Chad and Senegal (0.2), and equal to that of Burkina Faso, Cote d’Ivoire, Guinea, Kenya, Mauritania, Niger, Nigeria, and Sierra Leone. Europe has 11.5 per 100,000.^19^

Compared with 63% in Ethiopia, 48% (256 of 537) of ICU beds in Kenya are reported to have ventilators.^20^ A study of 12 ICUs (public and private) in Uganda reported 58% had access to renal replacement therapy compared to 10% in Ethiopia, and 92% were equipped with defibrillators compared to 73% in Ethiopia.^21^ The same study reports pipeline oxygen in 83% ICUs, whereas no surveyed ICU in Ethiopia had piped oxygen. It is also pertinent to note the gaps in IPC; only 14% ICUs had a designated room for isolation, similar to 16% reported in Kenya, yet access to PPE was higher at 71% compared to 52% to 59% for gowns and masks in Kenya.^22^

Comparison of ICUs across countries or regions is hampered by the lack of generalizable definitions.^13^ This study adopted the 2017 WFSICCM consensus definitions and criteria for categorizing ICUs.^10^ Ethiopian facilities have minimal capacity to provide monitoring or organ support, evidenced by most units functioning at level 1 as per WFSICCM standards.^23,24^ There is an urgent need to address these shortcomings and increase the capacity of ICUs, at least to level 2 standards in each region. In the last decade, Ethiopia has made significant efforts to improve perioperative capacity^5^; however, minimal effort has thus far been made to equip facilities with improved or level 3 critical care facilities to accompany the rise in surgical provision.

To do so will require additional resources; shortages of medical equipment often arise from a lack of biomedical support or replacement supplies. In Ethiopia, hospitals have individual budgets, determined by corresponding regional health agencies. ICU budgets, set by hospital management, are usually included within another budget, for example, internal medicine. Only 3 facilities had a dedicated ICU budget. Some larger supplies, for example, beds, ventilators, and monitoring devices are obtained and distributed by the MOH. A national strategy for supply chain management may help to address resource shortages and ensure equitable distribution.

By definition, an ICU is a place where patients are closely monitored and comprehensive support for failing organs is provided. Our study reports that Ethiopia has very limited capacity for mechanical ventilation and renal support. That less than a third of the ICU beds had the 3 most basic noninvasive monitoring devices is further stark evidence of the gaps in infrastructure. The inability to provide organ support needs correction by the immediate provision of an adequate and reliable oxygen supply, and the provision of equipment to provide basic monitoring and organ support. The MOH and professional societies in emergency medicine and critical care should stipulate standards for ICUs adapted for the Ethiopian setting, and support units to reach that standard. It is disappointing but not surprising that the vast majority of units had no access to audit, research, or quality improvement activities. The implementation of digital setting-adapted registry platforms in perioperative and critical care, enabling continuous data for audit, quality improvement, and research can address this gap.^25^

There has been appreciable investment in tertiary and supraspecialty care in Ethiopia over the last decade with increased access to surgery including specialized services such as renal transplantation and neurosurgical procedures, and the expansion of oncology care and treatment centers. The outcome of such investment is dependent on adequate and equitably distributed intensive care services in the country.

### Limitations of This Study

This study did not include private sector ICUs due to difficulties with access. Two large public ICUs treating COVID-19 patients exclusively were excluded for safety reasons. The status of these units was dynamic, with additional resources to support the pandemic response. This study therefore does not assume a national view. In addition, the reported confidence intervals do not include adjustment for within-hospital correlation. Incomplete data exist for IPC measures where data could not be verified; IPC is a sensitive subject area owing to pandemic-driven heightened publicity. With budget reallocations and efforts underway to improve critical care capacity in response to the pandemic, we recommend reassessment in the postpandemic era.

## RECOMMENDATIONS

This study highlights deficiencies in quantity, distribution, organization, and provision of intensive care in Ethiopia. The provision of adequate and reliable oxygen and basic monitoring for each ICU bed in Ethiopia is an urgent national priority, in parallel to any ICU expansion efforts. The MOH and professional bodies should also agree setting-adapted national minimum standards for ICUs. The implementation of setting-adapted, accessible, and continuous digital surveillance systems to aid quality evaluation and improvement should be encouraged. A holistic data-driven and multipronged initiative is required to improve the quality of critical care at regional and national level.

## ACKNOWLEDGMENTS

The authors wish to thank all hospital CEO’s and medical directors for their honest and cooperative response, and data collectors and coordinators who visited facilities for their assistance with data collection. We thank Ermiyas Belay, MSc, from Wolkite University, Ethiopia, and Dilanthi Gamage from Network for Improving Critical Care Systems and Training (NICST), Sri Lanka for their assistance in analyzing the data. We are particularly grateful to Prof Bruce Biccard, PhD, from University of Cape Town for his assistance in presubmission manuscript review.

## DISCLOSURES

**Name:** Fitsum Kifle, MSc.

**Contribution:** This author helped with conceptualization, methodology, analysis, and preparation of the manuscript.

**Name:** Yared Boru, MD.

**Contribution:** This author helped with conceptualization, methodology, project administration, analysis, and review and editing of the manuscript.

**Name:** Hailu Dhufera Tamiru, MD.

**Contribution:** This author helped with conceptualization, methodology, resources, analysis, and review and editing of the manuscript.

**Name:** Menbeu Sultan, MD.

**Contribution:** This author helped with conceptualization, methodology, supervision, analysis, review and editing of the manuscript.

**Name:** Yenegeta Walelign, MSc.

**Contribution:** This author helped with project administration, data acquisition, and approval of the final manuscript.

**Name:** Azeb Demelash, MSc.

**Contribution:** This author helped with project administration, data acquisition, and approval of the final manuscript.

**Name:** Abigail Beane, PhD.

**Contribution:** This author helped with interpretation of data, preparation of the manuscript, and approval of the final manuscript.

**Name:** Rashan Haniffa, PhD.

**Contribution:** This author helped with interpretation of data, preparation of the manuscript, and approval of the final manuscript.

**Name:** Alegnta Gebreyesus, MD.

**Contribution:** This author helped with conceptualization, methodology, supervision, and funding acquisition.

**Name:** Jolene Moore, MBChB.

**Contribution:** This author helped with visualization, analysis, and preparation of the manuscript.

**This manuscript was handled by:** Angela Enright, MB, FRCPC.
